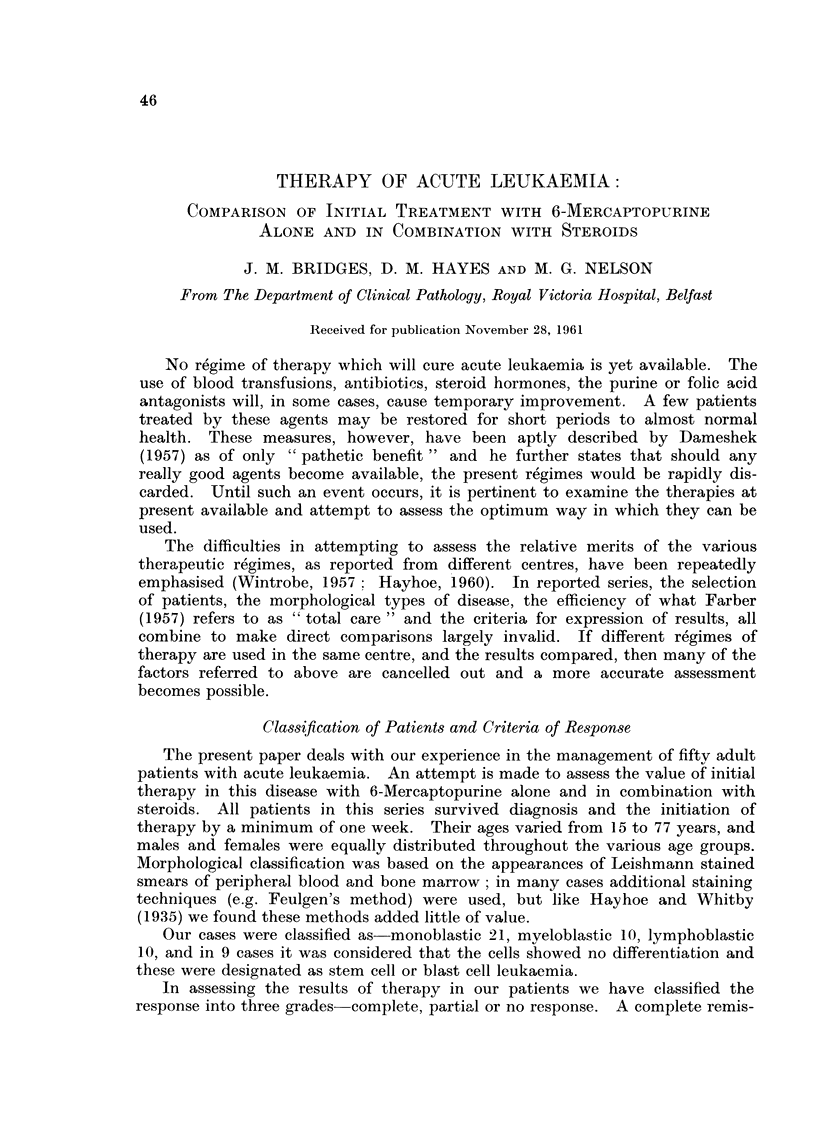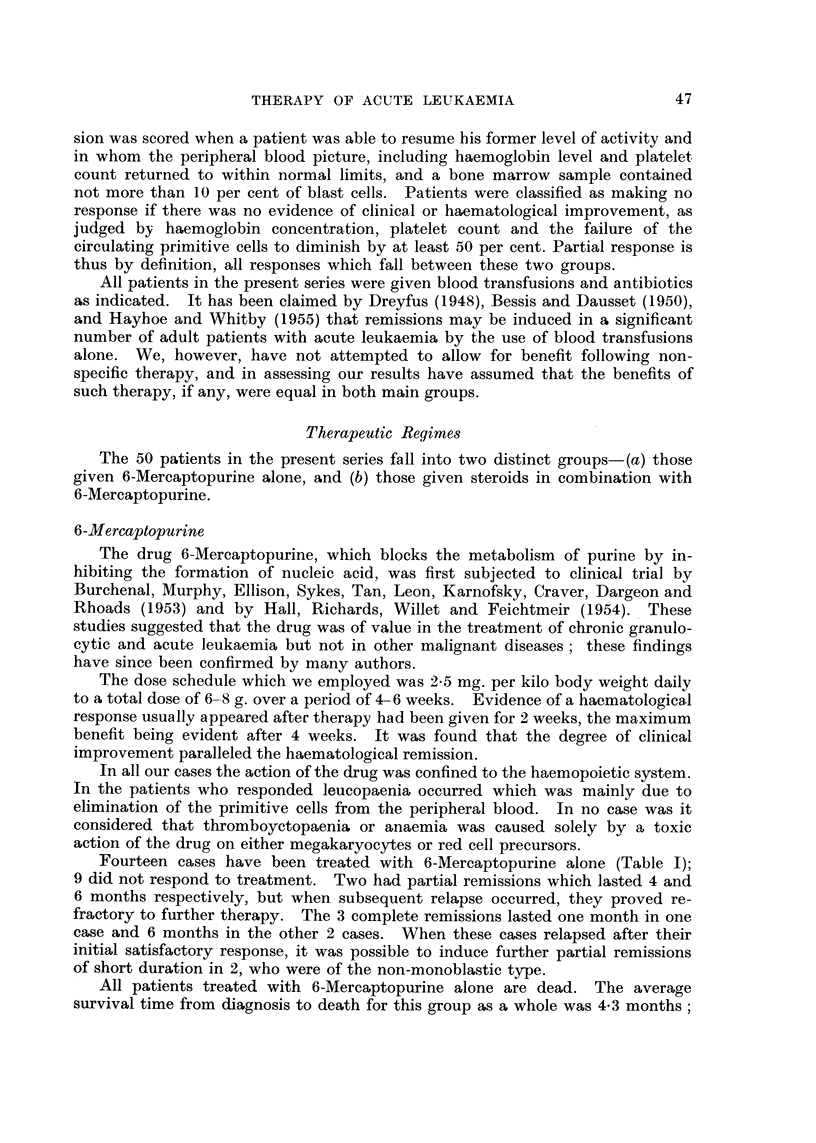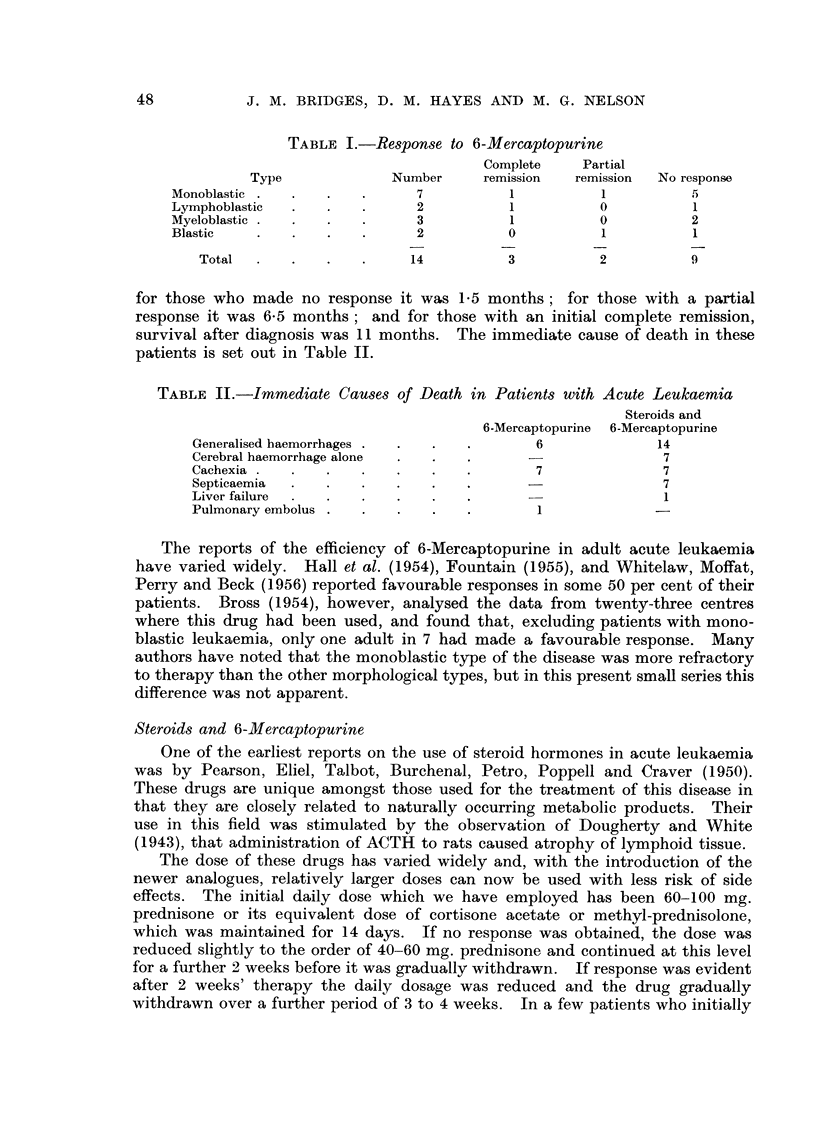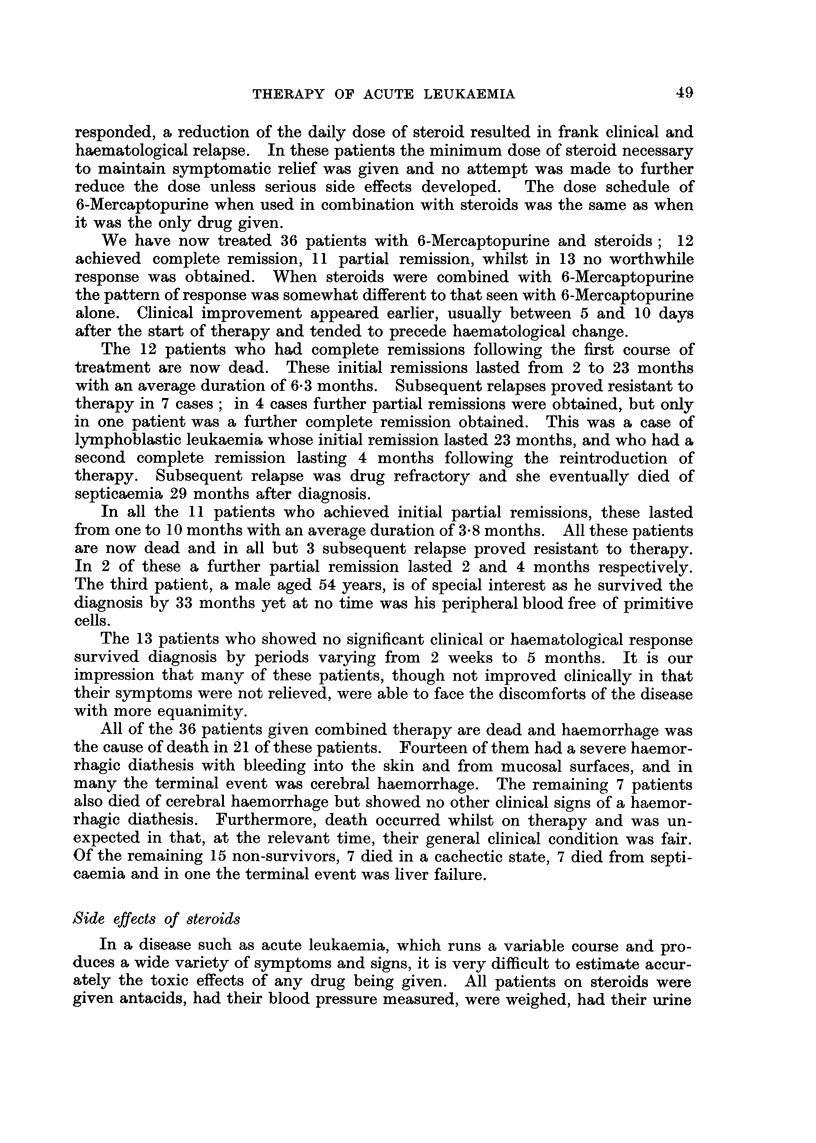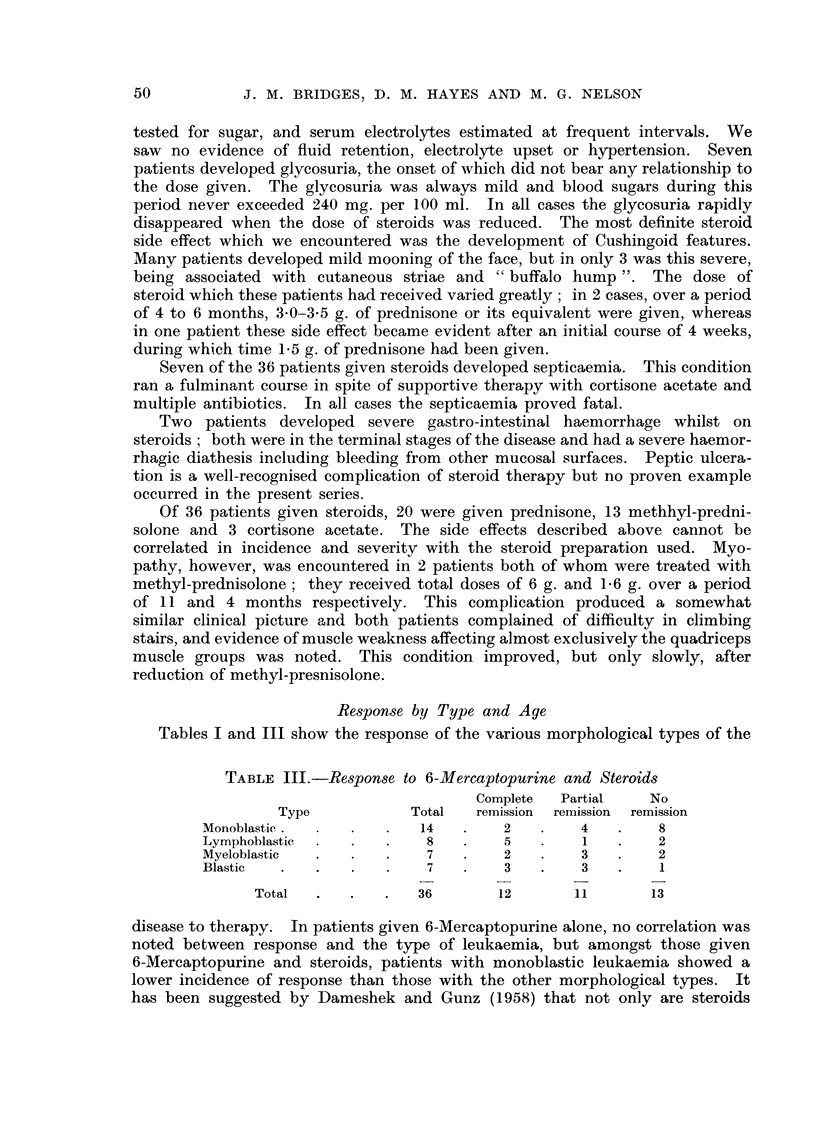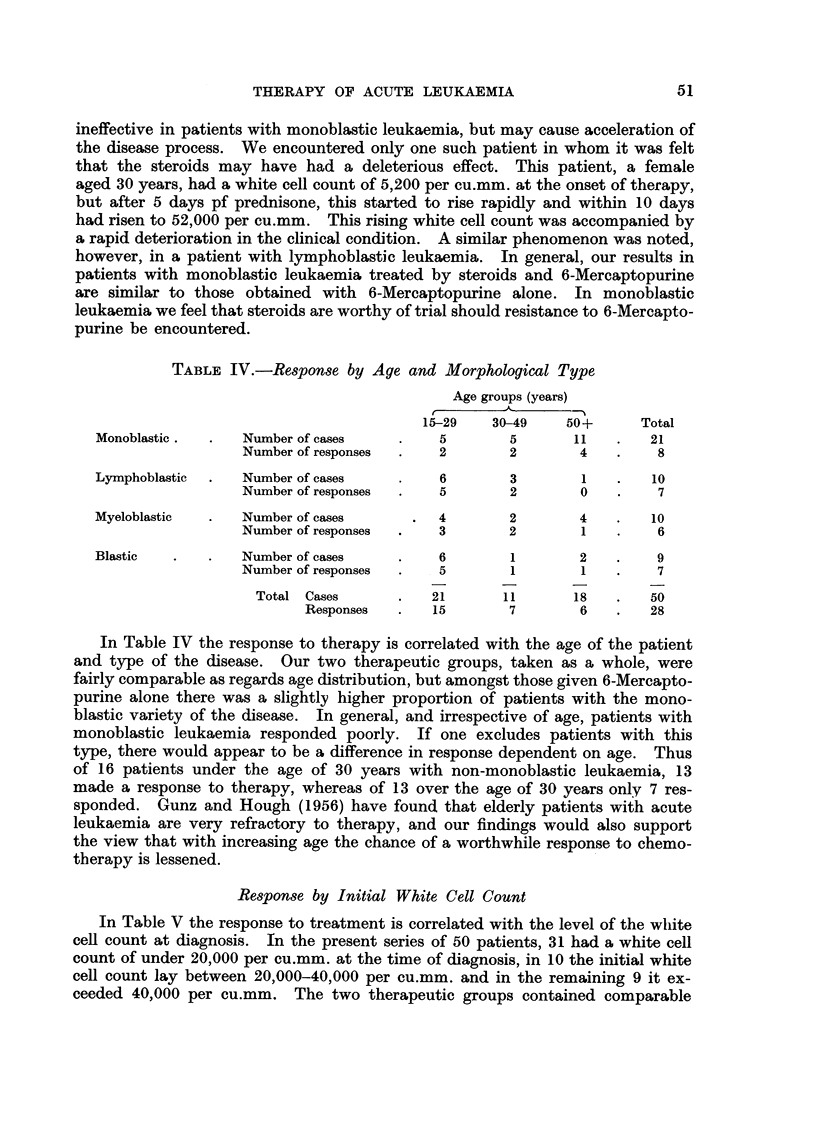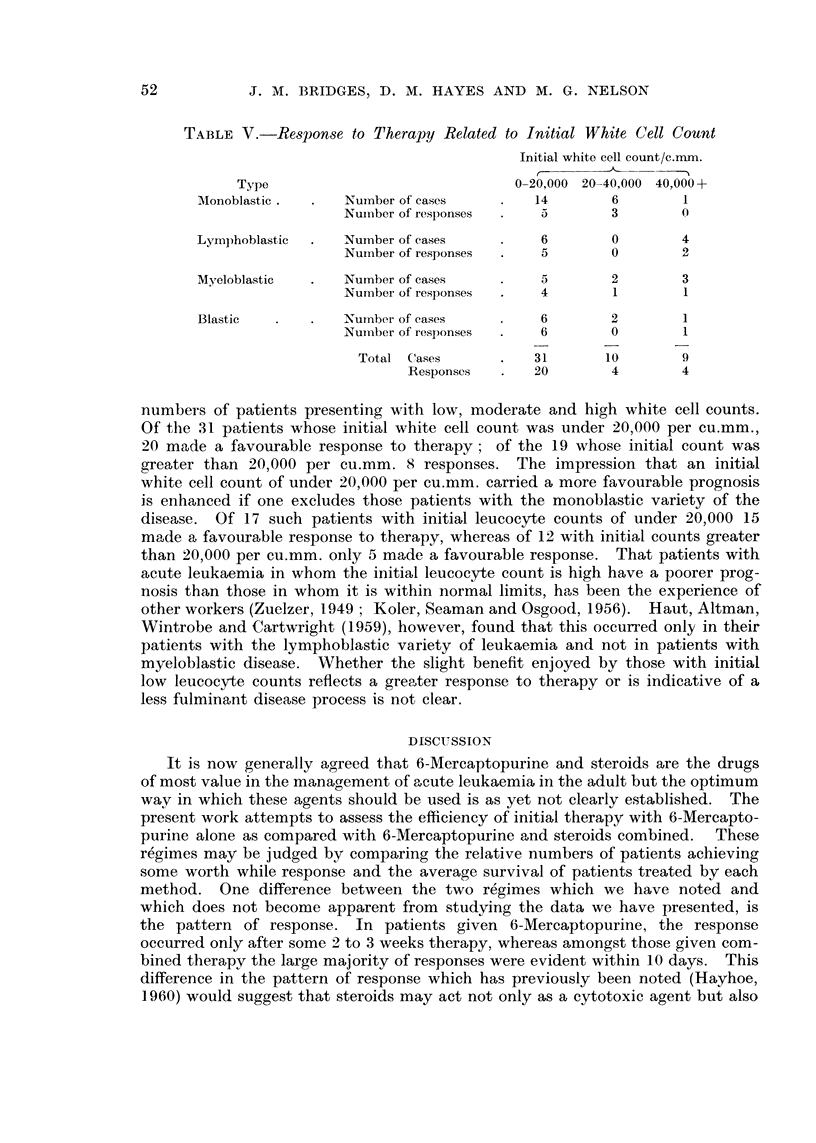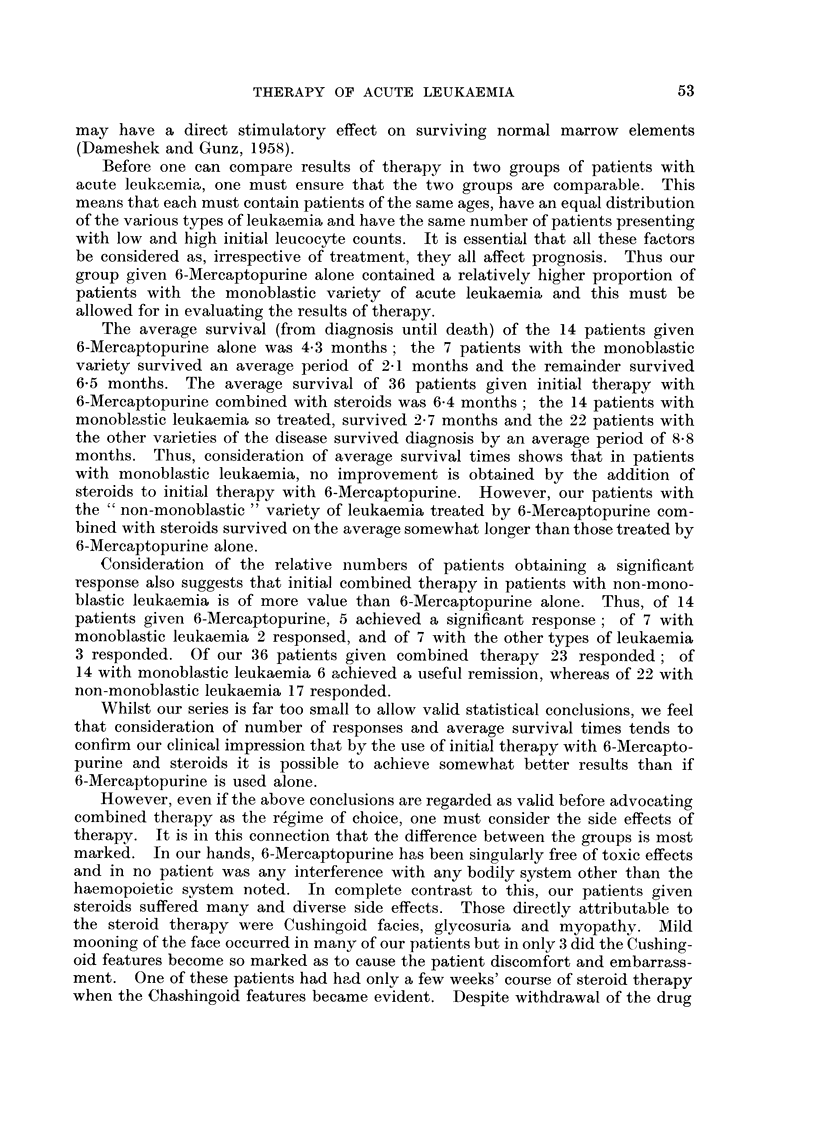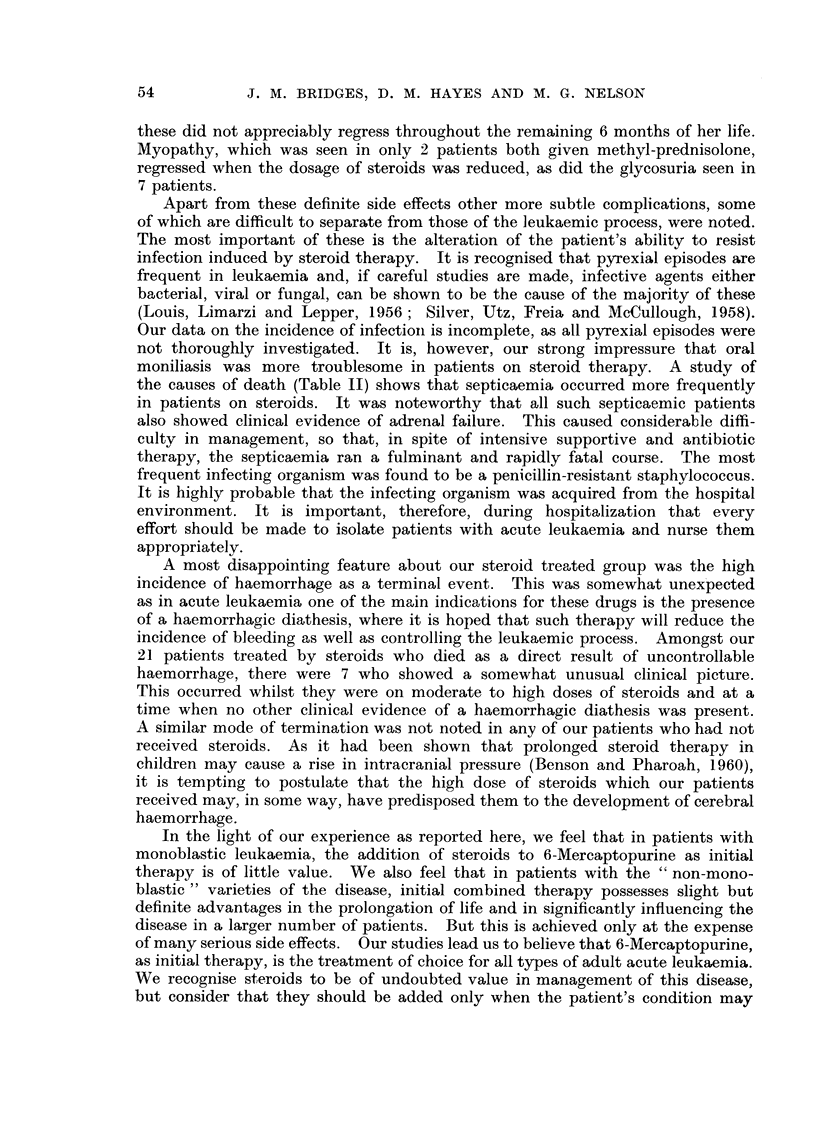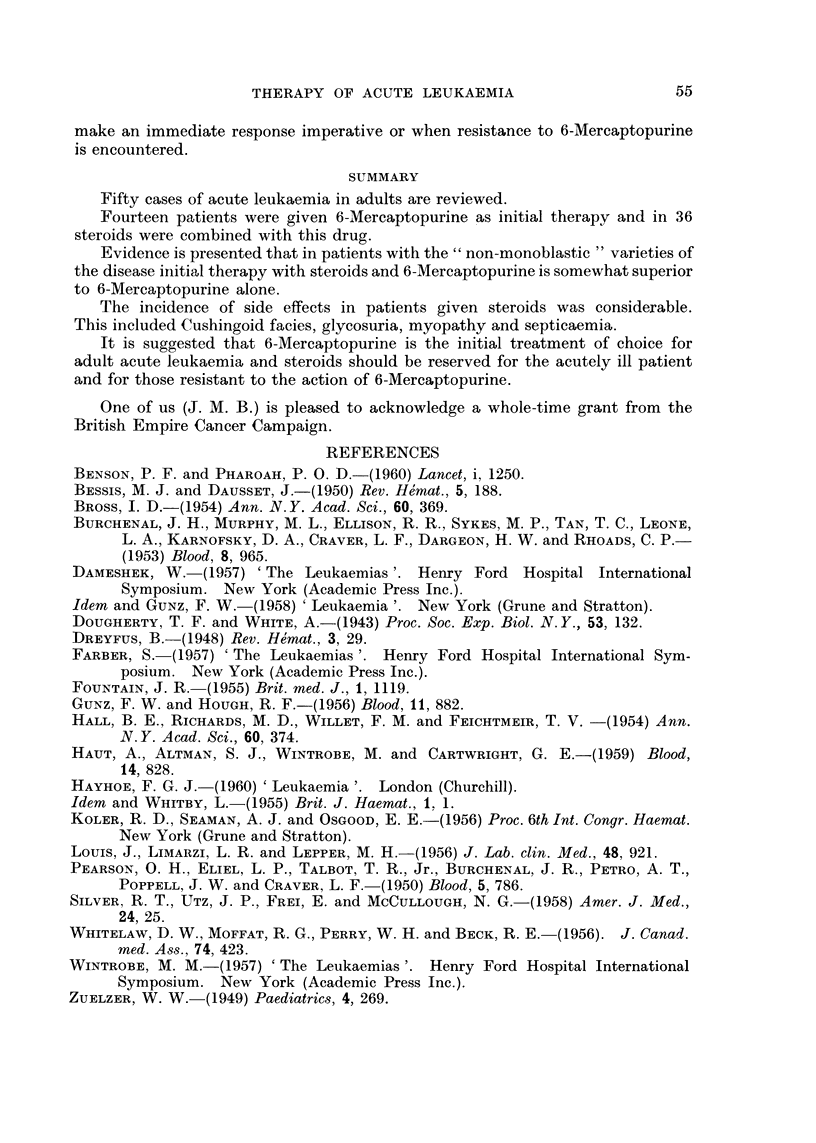# Therapy of Acute Leukaemia

**DOI:** 10.1038/bjc.1962.5

**Published:** 1962-03

**Authors:** J. M. Bridges, D. M. Hayes, M. G. Nelson


### THERAPY OF ACUTE LEUKAEMIA:

### Comparison of Initial Treatment with 6-Mercaptopurine Alone and in Combination with Steroids

J. M. BRIDGES, D. M. HAYES and M. G. NELSON

*From The Department of Clinical Pathology, Royal Victoria Hospital, Belfast*

Received for publication November 28, 1961

No régime of therapy which will cure acute leukaemia is yet available. The use of blood transfusions, antibiotics, steroid hormones, the purine or folic acid antagonists will, in some cases, cause temporary improvement. A few patients treated by these agents may be restored for short periods to almost normal health. These measures, however, have been aptly described by Dameshek (1957) as of only " pathetic benefit " and he further states that should any really good agents become available, the present régimes would be rapidly discarded. Until such an event occurs, it is pertinent to examine the therapies at present available and attempt to assess the optimum way in which they can be used.

The difficulties in attempting to assess the relative merits of the various therapeutic régimes, as reported from different centres, have been repeatedly emphasised (Wintrobe, 1957; Hayhoe, 1960). In reported series, the selection of patients, the morphological types of disease, the efficiency of what Farber (1957) refers to as " total care " and the criteria for expression of results, all combine to make direct comparisons largely invalid. If different régimes of therapy are used in the same centre, and the results compared, then many of the factors referred to above are cancelled out and a more accurate assessment becomes possible.

### *Classification of Patients and Criteria of Response*

The present paper deals with our experience in the management of fifty adult patients with acute leukaemia. An attempt is made to assess the value of initial therapy in this disease with 6-Mercaptopurine alone and in combination with steroids. All patients in this series survived diagnosis and the initiation of therapy by a minimum of one week. Their ages varied from 15 to 77 years, and males and females were equally distributed throughout the various age groups. Morphological classification was based on the appearances of Leishmann stained smears of peripheral blood and bone marrow ; in many cases additional staining techniques (e.g. Feulgen's method) were used, but like Hayhoe and Whitby (1935) we found these methods added little of value.

Our cases were classified as—monoblastic 21, myeloblastic 10, lymphoblastic 10, and in 9 cases it was considered that the cells showed no differentiation and these were designated as stem cell or blast cell leukaemia.

In assessing the results of therapy in our patients we have classified the response into three grades—complete, partial or no response. A complete remission was scored when a patient was able to resume his former level of activity and in whom the peripheral blood picture, including haemoglobin level and platelet count returned to within normal limits, and a bone marrow sample contained not more than 10 per cent of blast cells. Patients were classified as making no response if there was no evidence of clinical or haematological improvement, as judged by haemoglobin concentration, platelet count and the failure of the circulating primitive cells to diminish by at least 50 per cent. Partial response is thus by definition, all responses which fall between these two groups.

All patients in the present series were given blood transfusions and antibiotics as indicated. It has been claimed by Dreyfus (1948), Bessis and Dausset (1950), and Hayhoe and Whitby (1955) that remissions may be induced in a significant number of adult patients with acute leukaemia by the use of blood transfusions alone. We, however, have not attempted to allow for benefit following non-specific therapy, and in assessing our results have assumed that the benefits of such therapy, if any, were equal in both main groups.

### *Therapeutic Regimes*

The 50 patients in the present series fall into two distinct groups—(*a*) those given 6-Mercaptopurine alone, and (*b*) those given steroids in combination with 6-Mercaptopurine.

#### 6-*Mercaptopurine*

The drug 6-Mercaptopurine, which blocks the metabolism of purine by inhibiting the formation of nucleic acid, was first subjected to clinical trial by Burchenal, Murphy, Ellison, Sykes, Tan, Leon, Karnofsky, Craver, Dargeon and Rhoads (1953) and by Hall, Richards, Willet and Feichtmeir (1954). These studies suggested that the drug was of value in the treatment of chronic granulocytic and acute leukaemia but not in other malignant diseases ; these findings have since been confirmed by many authors.

The dose schedule which we employed was 2·5 mg. per kilo body weight daily to a total dose of 6–8 g. over a period of 4–6 weeks. Evidence of a haematological response usually appeared after therapy had been given for 2 weeks, the maximum benefit being evident after 4 weeks. It was found that the degree of clinical improvement paralleled the haematological remission.

In all our cases the action of the drug was confined to the haemopoietic system. In the patients who responded leucopaenia occurred which was mainly due to elimination of the primitive cells from the peripheral blood. In no case was it considered that thromboyctopaenia or anaemia was caused solely by a toxic action of the drug on either megakaryocytes or red cell precursors.

Fourteen cases have been treated with 6-Mercaptopurine alone (Table I); 9 did not respond to treatment. Two had partial remissions which lasted 4 and 6 months respectively, but when subsequent relapse occurred, they proved refractory to further therapy. The 3 complete remissions lasted one month in one case and 6 months in the other 2 cases. When these cases relapsed after their initial satisfactory response, it was possible to induce further partial remissions of short duration in 2, who were of the non-monoblastic type.

All patients treated with 6-Mercaptopurine alone are dead. The average survival time from diagnosis to death for this group as a whole was 4·3 months ;

TABLE I.—*Response to 6-Mercaptopurine*

| Type | Number | Complete remission | Partial remission | No response |
|---|---|---|---|---|
| Monoblastic . . . . | 7 | 1 | 1 | 5 |
| Lymphoblastic . . . | 2 | 1 | 0 | 1 |
| Myeloblastic . . . . | 3 | 1 | 0 | 2 |
| Blastic . . . . | 2 | 0 | 1 | 1 |
| Total . . . . | 14 | 3 | 2 | 9 |

for those who made no response it was 1·5 months ; for those with a partial response it was 6·5 months ; and for those with an initial complete remission, survival after diagnosis was 11 months. The immediate cause of death in these patients is set out in Table II.

TABLE II.—*Immediate Causes of Death in Patients with Acute Leukaemia*

| | 6-Mercaptopurine | Steroids and 6-Mercaptopurine |
|---|---|---|
| Generalised haemorrhages . . . . | 6 | 14 |
| Cerebral haemorrhage alone . . . | — | 7 |
| Cachexia . . . . . . . . | 7 | 7 |
| Septicaemia . . . . . . . | — | 7 |
| Liver failure . . . . . . | — | 1 |
| Pulmonary embolus . . . . . | 1 | — |

The reports of the efficiency of 6-Mercaptopurine in adult acute leukaemia have varied widely. Hall *et al.* (1954), Fountain (1955), and Whitelaw, Moffat, Perry and Beck (1956) reported favourable responses in some 50 per cent of their patients. Bross (1954), however, analysed the data from twenty-three centres where this drug had been used, and found that, excluding patients with monoblastic leukaemia, only one adult in 7 had made a favourable response. Many authors have noted that the monoblastic type of the disease was more refractory to therapy than the other morphological types, but in this present small series this difference was not apparent.

*Steroids and 6-Mercaptopurine*

One of the earliest reports on the use of steroid hormones in acute leukaemia was by Pearson, Eliel, Talbot, Burchenal, Petro, Poppell and Craver (1950). These drugs are unique amongst those used for the treatment of this disease in that they are closely related to naturally occurring metabolic products. Their use in this field was stimulated by the observation of Dougherty and White (1943), that administration of ACTH to rats caused atrophy of lymphoid tissue.

The dose of these drugs has varied widely and, with the introduction of the newer analogues, relatively larger doses can now be used with less risk of side effects. The initial daily dose which we have employed has been 60–100 mg. prednisone or its equivalent dose of cortisone acetate or methyl-prednisolone, which was maintained for 14 days. If no response was obtained, the dose was reduced slightly to the order of 40–60 mg. prednisone and continued at this level for a further 2 weeks before it was gradually withdrawn. If response was evident after 2 weeks' therapy the daily dosage was reduced and the drug gradually withdrawn over a further period of 3 to 4 weeks. In a few patients who initially responded, a reduction of the daily dose of steroid resulted in frank clinical and haematological relapse. In these patients the minimum dose of steroid necessary to maintain symptomatic relief was given and no attempt was made to further reduce the dose unless serious side effects developed. The dose schedule of 6-Mercaptopurine when used in combination with steroids was the same as when it was the only drug given.

We have now treated 36 patients with 6-Mercaptopurine and steroids; 12 achieved complete remission, 11 partial remission, whilst in 13 no worthwhile response was obtained. When steroids were combined with 6-Mercaptopurine the pattern of response was somewhat different to that seen with 6-Mercaptopurine alone. Clinical improvement appeared earlier, usually between 5 and 10 days after the start of therapy and tended to precede haematological change.

The 12 patients who had complete remissions following the first course of treatment are now dead. These initial remissions lasted from 2 to 23 months with an average duration of 6·3 months. Subsequent relapses proved resistant to therapy in 7 cases; in 4 cases further partial remissions were obtained, but only in one patient was a further complete remission obtained. This was a case of lymphoblastic leukaemia whose initial remission lasted 23 months, and who had a second complete remission lasting 4 months following the reintroduction of therapy. Subsequent relapse was drug refractory and she eventually died of septicaemia 29 months after diagnosis.

In all the 11 patients who achieved initial partial remissions, these lasted from one to 10 months with an average duration of 3·8 months. All these patients are now dead and in all but 3 subsequent relapse proved resistant to therapy. In 2 of these a further partial remission lasted 2 and 4 months respectively. The third patient, a male aged 54 years, is of special interest as he survived the diagnosis by 33 months yet at no time was his peripheral blood free of primitive cells.

The 13 patients who showed no significant clinical or haematological response survived diagnosis by periods varying from 2 weeks to 5 months. It is our impression that many of these patients, though not improved clinically in that their symptoms were not relieved, were able to face the discomforts of the disease with more equanimity.

All of the 36 patients given combined therapy are dead and haemorrhage was the cause of death in 21 of these patients. Fourteen of them had a severe haemorrhagic diathesis with bleeding into the skin and from mucosal surfaces, and in many the terminal event was cerebral haemorrhage. The remaining 7 patients also died of cerebral haemorrhage but showed no other clinical signs of a haemorrhagic diathesis. Furthermore, death occurred whilst on therapy and was unexpected in that, at the relevant time, their general clinical condition was fair. Of the remaining 15 non-survivors, 7 died in a cachectic state, 7 died from septicaemia and in one the terminal event was liver failure.

*Side effects of steroids*

In a disease such as acute leukaemia, which runs a variable course and produces a wide variety of symptoms and signs, it is very difficult to estimate accurately the toxic effects of any drug being given. All patients on steroids were given antacids, had their blood pressure measured, were weighed, had their urine tested for sugar, and serum electrolytes estimated at frequent intervals. We saw no evidence of fluid retention, electrolyte upset or hypertension. Seven patients developed glycosuria, the onset of which did not bear any relationship to the dose given. The glycosuria was always mild and blood sugars during this period never exceeded 240 mg. per 100 ml. In all cases the glycosuria rapidly disappeared when the dose of steroids was reduced. The most definite steroid side effect which we encountered was the development of Cushingoid features. Many patients developed mild mooning of the face, but in only 3 was this severe, being associated with cutaneous striae and " buffalo hump ". The dose of steroid which these patients had received varied greatly ; in 2 cases, over a period of 4 to 6 months, 3·0–3·5 g. of prednisone or its equivalent were given, whereas in one patient these side effect became evident after an initial course of 4 weeks, during which time 1·5 g. of prednisone had been given.

Seven of the 36 patients given steroids developed septicaemia. This condition ran a fulminant course in spite of supportive therapy with cortisone acetate and multiple antibiotics. In all cases the septicaemia proved fatal.

Two patients developed severe gastro-intestinal haemorrhage whilst on steroids ; both were in the terminal stages of the disease and had a severe haemorrhagic diathesis including bleeding from other mucosal surfaces. Peptic ulceration is a well-recognised complication of steroid therapy but no proven example occurred in the present series.

Of 36 patients given steroids, 20 were given prednisone, 13 methhyl-prednisolone and 3 cortisone acetate. The side effects described above cannot be correlated in incidence and severity with the steroid preparation used. Myopathy, however, was encountered in 2 patients both of whom were treated with methyl-prednisolone ; they received total doses of 6 g. and 1·6 g. over a period of 11 and 4 months respectively. This complication produced a somewhat similar clinical picture and both patients complained of difficulty in climbing stairs, and evidence of muscle weakness affecting almost exclusively the quadriceps muscle groups was noted. This condition improved, but only slowly, after reduction of methyl-presnisolone.

*Response by Type and Age*

Tables I and III show the response of the various morphological types of the disease to therapy. In patients given 6-Mercaptopurine alone, no correlation was noted between response and the type of leukaemia, but amongst those given 6-Mercaptopurine and steroids, patients with monoblastic leukaemia showed a lower incidence of response than those with the other morphological types. It has been suggested by Dameshek and Gunz (1958) that not only are steroids

TABLE III.—*Response to 6-Mercaptopurine and Steroids*

| Type | Total | Complete remission | Partial remission | No remission |
|---|---|---|---|---|
| Monoblastic | 14 | 2 | 4 | 8 |
| Lymphoblastic | 8 | 5 | 1 | 2 |
| Myeloblastic | 7 | 2 | 3 | 2 |
| Blastic | 7 | 3 | 3 | 1 |
| Total | 36 | 12 | 11 | 13 |

ineffective in patients with monoblastic leukaemia, but may cause acceleration of the disease process. We encountered only one such patient in whom it was felt that the steroids may have had a deleterious effect. This patient, a female aged 30 years, had a white cell count of 5,200 per cu.mm. at the onset of therapy, but after 5 days pf prednisone, this started to rise rapidly and within 10 days had risen to 52,000 per cu.mm. This rising white cell count was accompanied by a rapid deterioration in the clinical condition. A similar phenomenon was noted, however, in a patient with lymphoblastic leukaemia. In general, our results in patients with monoblastic leukaemia treated by steroids and 6-Mercaptopurine are similar to those obtained with 6-Mercaptopurine alone. In monoblastic leukaemia we feel that steroids are worthy of trial should resistance to 6-Mercaptopurine be encountered.

TABLE IV.—*Response by Age and Morphological Type*

| | | Age groups (years) | | | |
|---|---|---|---|---|---|
| | | 15–29 | 30–49 | 50+ | Total |
| Monoblastic | Number of cases | 5 | 5 | 11 | 21 |
| | Number of responses | 2 | 2 | 4 | 8 |
| Lymphoblastic | Number of cases | 6 | 3 | 1 | 10 |
| | Number of responses | 5 | 2 | 0 | 7 |
| Myeloblastic | Number of cases | 4 | 2 | 4 | 10 |
| | Number of responses | 3 | 2 | 1 | 6 |
| Blastic | Number of cases | 6 | 1 | 2 | 9 |
| | Number of responses | 5 | 1 | 1 | 7 |
| Total | Cases | 21 | 11 | 18 | 50 |
| | Responses | 15 | 7 | 6 | 28 |

In Table IV the response to therapy is correlated with the age of the patient and type of the disease. Our two therapeutic groups, taken as a whole, were fairly comparable as regards age distribution, but amongst those given 6-Mercaptopurine alone there was a slightly higher proportion of patients with the monoblastic variety of the disease. In general, and irrespective of age, patients with monoblastic leukaemia responded poorly. If one excludes patients with this type, there would appear to be a difference in response dependent on age. Thus of 16 patients under the age of 30 years with non-monoblastic leukaemia, 13 made a response to therapy, whereas of 13 over the age of 30 years only 7 responded. Gunz and Hough (1956) have found that elderly patients with acute leukaemia are very refractory to therapy, and our findings would also support the view that with increasing age the chance of a worthwhile response to chemotherapy is lessened.

### *Response by Initial White Cell Count*

In Table V the response to treatment is correlated with the level of the white cell count at diagnosis. In the present series of 50 patients, 31 had a white cell count of under 20,000 per cu.mm. at the time of diagnosis, in 10 the initial white cell count lay between 20,000–40,000 per cu.mm. and in the remaining 9 it exceeded 40,000 per cu.mm. The two therapeutic groups contained comparable

TABLE V.—*Response to Therapy Related to Initial White Cell Count*

| Type | | Initial white cell count/c.mm. 0–20,000 | 20–40,000 | 40,000+ |
|---|---|---|---|---|
| Monoblastic | Number of cases | 14 | 6 | 1 |
| | Number of responses | 5 | 3 | 0 |
| Lymphoblastic | Number of cases | 6 | 0 | 4 |
| | Number of responses | 5 | 0 | 2 |
| Myeloblastic | Number of cases | 5 | 2 | 3 |
| | Number of responses | 4 | 1 | 1 |
| Blastic | Number of cases | 6 | 2 | 1 |
| | Number of responses | 6 | 0 | 1 |
| Total | Cases | 31 | 10 | 9 |
| | Responses | 20 | 4 | 4 |

numbers of patients presenting with low, moderate and high white cell counts. Of the 31 patients whose initial white cell count was under 20,000 per cu.mm., 20 made a favourable response to therapy ; of the 19 whose initial count was greater than 20,000 per cu.mm. 8 responses. The impression that an initial white cell count of under 20,000 per cu.mm. carried a more favourable prognosis is enhanced if one excludes those patients with the monoblastic variety of the disease. Of 17 such patients with initial leucocyte counts of under 20,000 15 made a favourable response to therapy, whereas of 12 with initial counts greater than 20,000 per cu.mm. only 5 made a favourable response. That patients with acute leukaemia in whom the initial leucocyte count is high have a poorer prognosis than those in whom it is within normal limits, has been the experience of other workers (Zuelzer, 1949 ; Koler, Seaman and Osgood, 1956). Haut, Altman, Wintrobe and Cartwright (1959), however, found that this occurred only in their patients with the lymphoblastic variety of leukaemia and not in patients with myeloblastic disease. Whether the slight benefit enjoyed by those with initial low leucocyte counts reflects a greater response to therapy or is indicative of a less fulminant disease process is not clear.

### DISCUSSION

It is now generally agreed that 6-Mercaptopurine and steroids are the drugs of most value in the management of acute leukaemia in the adult but the optimum way in which these agents should be used is as yet not clearly established. The present work attempts to assess the efficiency of initial therapy with 6-Mercaptopurine alone as compared with 6-Mercaptopurine and steroids combined. These régimes may be judged by comparing the relative numbers of patients achieving some worth while response and the average survival of patients treated by each method. One difference between the two régimes which we have noted and which does not become apparent from studying the data we have presented, is the pattern of response. In patients given 6-Mercaptopurine, the response occurred only after some 2 to 3 weeks therapy, whereas amongst those given combined therapy the large majority of responses were evident within 10 days. This difference in the pattern of response which has previously been noted (Hayhoe, 1960) would suggest that steroids may act not only as a cytotoxic agent but also may have a direct stimulatory effect on surviving normal marrow elements (Dameshek and Gunz, 1958).

Before one can compare results of therapy in two groups of patients with acute leukaemia, one must ensure that the two groups are comparable. This means that each must contain patients of the same ages, have an equal distribution of the various types of leukaemia and have the same number of patients presenting with low and high initial leucocyte counts. It is essential that all these factors be considered as, irrespective of treatment, they all affect prognosis. Thus our group given 6-Mercaptopurine alone contained a relatively higher proportion of patients with the monoblastic variety of acute leukaemia and this must be allowed for in evaluating the results of therapy.

The average survival (from diagnosis until death) of the 14 patients given 6-Mercaptopurine alone was 4·3 months ; the 7 patients with the monoblastic variety survived an average period of 2·1 months and the remainder survived 6·5 months. The average survival of 36 patients given initial therapy with 6-Mercaptopurine combined with steroids was 6·4 months ; the 14 patients with monoblastic leukaemia so treated, survived 2·7 months and the 22 patients with the other varieties of the disease survived diagnosis by an average period of 8·8 months. Thus, consideration of average survival times shows that in patients with monoblastic leukaemia, no improvement is obtained by the addition of steroids to initial therapy with 6-Mercaptopurine. However, our patients with the " non-monoblastic " variety of leukaemia treated by 6-Mercaptopurine combined with steroids survived on the average somewhat longer than those treated by 6-Mercaptopurine alone.

Consideration of the relative numbers of patients obtaining a significant response also suggests that initial combined therapy in patients with non-monoblastic leukaemia is of more value than 6-Mercaptopurine alone. Thus, of 14 patients given 6-Mercaptopurine, 5 achieved a significant response ; of 7 with monoblastic leukaemia 2 responsed, and of 7 with the other types of leukaemia 3 responded. Of our 36 patients given combined therapy 23 responded ; of 14 with monoblastic leukaemia 6 achieved a useful remission, whereas of 22 with non-monoblastic leukaemia 17 responded.

Whilst our series is far too small to allow valid statistical conclusions, we feel that consideration of number of responses and average survival times tends to confirm our clinical impression that by the use of initial therapy with 6-Mercaptopurine and steroids it is possible to achieve somewhat better results than if 6-Mercaptopurine is used alone.

However, even if the above conclusions are regarded as valid before advocating combined therapy as the régime of choice, one must consider the side effects of therapy. It is in this connection that the difference between the groups is most marked. In our hands, 6-Mercaptopurine has been singularly free of toxic effects and in no patient was any interference with any bodily system other than the haemopoietic system noted. In complete contrast to this, our patients given steroids suffered many and diverse side effects. Those directly attributable to the steroid therapy were Cushingoid facies, glycosuria and myopathy. Mild mooning of the face occurred in many of our patients but in only 3 did the Cushingoid features become so marked as to cause the patient discomfort and embarrassment. One of these patients had had only a few weeks' course of steroid therapy when the Chashingoid features became evident. Despite withdrawal of the drug these did not appreciably regress throughout the remaining 6 months of her life. Myopathy, which was seen in only 2 patients both given methyl-prednisolone, regressed when the dosage of steroids was reduced, as did the glycosuria seen in 7 patients.

Apart from these definite side effects other more subtle complications, some of which are difficult to separate from those of the leukaemic process, were noted. The most important of these is the alteration of the patient's ability to resist infection induced by steroid therapy. It is recognised that pyrexial episodes are frequent in leukaemia and, if careful studies are made, infective agents either bacterial, viral or fungal, can be shown to be the cause of the majority of these (Louis, Limarzi and Lepper, 1956; Silver, Utz, Freia and McCullough, 1958). Our data on the incidence of infection is incomplete, as all pyrexial episodes were not thoroughly investigated. It is, however, our strong impressure that oral moniliasis was more troublesome in patients on steroid therapy. A study of the causes of death (Table II) shows that septicaemia occurred more frequently in patients on steroids. It was noteworthy that all such septicaemic patients also showed clinical evidence of adrenal failure. This caused considerable difficulty in management, so that, in spite of intensive supportive and antibiotic therapy, the septicaemia ran a fulminant and rapidly fatal course. The most frequent infecting organism was found to be a penicillin-resistant staphylococcus. It is highly probable that the infecting organism was acquired from the hospital environment. It is important, therefore, during hospitalization that every effort should be made to isolate patients with acute leukaemia and nurse them appropriately.

A most disappointing feature about our steroid treated group was the high incidence of haemorrhage as a terminal event. This was somewhat unexpected as in acute leukaemia one of the main indications for these drugs is the presence of a haemorrhagic diathesis, where it is hoped that such therapy will reduce the incidence of bleeding as well as controlling the leukaemic process. Amongst our 21 patients treated by steroids who died as a direct result of uncontrollable haemorrhage, there were 7 who showed a somewhat unusual clinical picture. This occurred whilst they were on moderate to high doses of steroids and at a time when no other clinical evidence of a haemorrhagic diathesis was present. A similar mode of termination was not noted in any of our patients who had not received steroids. As it had been shown that prolonged steroid therapy in children may cause a rise in intracranial pressure (Benson and Pharoah, 1960), it is tempting to postulate that the high dose of steroids which our patients received may, in some way, have predisposed them to the development of cerebral haemorrhage.

In the light of our experience as reported here, we feel that in patients with monoblastic leukaemia, the addition of steroids to 6-Mercaptopurine as initial therapy is of little value. We also feel that in patients with the " non-monoblastic " varieties of the disease, initial combined therapy possesses slight but definite advantages in the prolongation of life and in significantly influencing the disease in a larger number of patients. But this is achieved only at the expense of many serious side effects. Our studies lead us to believe that 6-Mercaptopurine, as initial therapy, is the treatment of choice for all types of adult acute leukaemia. We recognise steroids to be of undoubted value in management of this disease, but consider that they should be added only when the patient's condition may make an immediate response imperative or when resistance to 6-Mercaptopurine is encountered.

SUMMARY

Fifty cases of acute leukaemia in adults are reviewed.

Fourteen patients were given 6-Mercaptopurine as initial therapy and in 36 steroids were combined with this drug.

Evidence is presented that in patients with the " non-monoblastic " varieties of the disease initial therapy with steroids and 6-Mercaptopurine is somewhat superior to 6-Mercaptopurine alone.

The incidence of side effects in patients given steroids was considerable. This included Cushingoid facies, glycosuria, myopathy and septicaemia.

It is suggested that 6-Mercaptopurine is the initial treatment of choice for adult acute leukaemia and steroids should be reserved for the acutely ill patient and for those resistant to the action of 6-Mercaptopurine.

One of us (J. M. B.) is pleased to acknowledge a whole-time grant from the British Empire Cancer Campaign.

REFERENCES

Benson, P. F. and Pharoah, P. O. D.—(1960) *Lancet*, i, 1250.

Bessis, M. J. and Dausset, J.—(1950) *Rev. Hémat.*, **5**, 188.

Bross, I. D.—(1954) *Ann. N.Y. Acad. Sci.*, **60**, 369.

Burchenal, J. H., Murphy, M. L., Ellison, R. R., Sykes, M. P., Tan, T. C., Leone, L. A., Karnofsky, D. A., Craver, L. F., Dargeon, H. W. and Rhoads, C. P.—(1953) *Blood*, **8**, 965.

Dameshek, W.—(1957) 'The Leukaemias'. Henry Ford Hospital International Symposium. New York (Academic Press Inc.).

*Idem* and Gunz, F. W.—(1958) 'Leukaemia'. New York (Grune and Stratton).

Dougherty, T. F. and White, A.—(1943) *Proc. Soc. Exp. Biol. N.Y.*, **53**, 132.

Dreyfus, B.—(1948) *Rev. Hémat.*, **3**, 29.

Farber, S.—(1957) 'The Leukaemias'. Henry Ford Hospital International Symposium. New York (Academic Press Inc.).

Fountain, J. R.—(1955) *Brit. med. J.*, **1**, 1119.

Gunz, F. W. and Hough, R. F.—(1956) *Blood*, **11**, 882.

Hall, B. E., Richards, M. D., Willet, F. M. and Feichtmeir, T. V. —(1954) *Ann. N.Y. Acad. Sci.*, **60**, 374.

Haut, A., Altman, S. J., Wintrobe, M. and Cartwright, G. E.—(1959) *Blood*, **14**, 828.

Hayhoe, F. G. J.—(1960) 'Leukaemia'. London (Churchill).

*Idem* and Whitby, L.—(1955) *Brit. J. Haemat.*, **1**, 1.

Koler, R. D., Seaman, A. J. and Osgood, E. E.—(1956) *Proc. 6th Int. Congr. Haemat.* New York (Grune and Stratton).

Louis, J., Limarzi, L. R. and Lepper, M. H.—(1956) *J. Lab. clin. Med.*, **48**, 921.

Pearson, O. H., Eliel, L. P., Talbot, T. R., Jr., Burchenal, J. R., Petro, A. T., Poppell, J. W. and Craver, L. F.—(1950) *Blood*, **5**, 786.

Silver, R. T., Utz, J. P., Frei, E. and McCullough, N. G.—(1958) *Amer. J. Med.*, **24**, 25.

Whitelaw, D. W., Moffat, R. G., Perry, W. H. and Beck, R. E.—(1956). *J. Canad. med. Ass.*, **74**, 423.

Wintrobe, M. M.—(1957) 'The Leukaemias'. Henry Ford Hospital International Symposium. New York (Academic Press Inc.).

Zuelzer, W. W.—(1949) *Paediatrics*, **4**, 269.